# To Defend or Not To Defend: That’s the Question

**DOI:** 10.1128/mSphere.00127-16

**Published:** 2016-06-01

**Authors:** Sriram Varahan, Lynn E. Hancock

**Affiliations:** a Institute for Stem Cell Biology and Regenerative Medicine (inStem), National Centre for Biological Sciences, Bangalore, India; b Department of Molecular Biosciences, University of Kansas, Lawrence, Kansas, USA

**Keywords:** CRISPR, *Enterococcus faecalis*, antibiotic resistance

## Abstract

*Enterococcus faecalis* is an opportunistic pathogen and is one of the leading causes of nosocomial infections. *E. faecalis* harbors a number of antibiotic resistance genes, and most of these are present on mobile genetic elements (MGEs) that can be disseminated within the species, as well as to other members of the human microflora. In an article by Price and colleagues [V. J. Price et al., mSphere 1(3):e00064-16, 2016, http://dx.doi.org/10.1128/mSphere.00064-16], the authors demonstrated how *E. faecalis* uses a restriction-modification system along with a clustered regularly interspaced short palindromic repeat (CRISPR)-Cas to function as a bacterial innate and adaptive immune system to regulate the influx of MGEs. The absence of these systems in high-risk hospital-adapted lineages of *E. faecalis*, including the prototypical V583 strain, appears to allow the ready acquisition of new traits that aid in the adaptation to new environmental stresses, including the evolution of resistance to many of our best antibiotics.

## COMMENTARY

The acquisition of mobile genetic elements (MGEs), constituting up to a quarter of the genome, has endowed the hospital-adapted lineages of *E. faecalis* with antibiotic resistance properties and virulence traits providing a selective advantage over their less-adapted counterparts under conditions of antibiotic selection for these strains (1). A study by Palmer and Gilmore (2) showed that the presence of MGEs in nearly all multidrug-resistant lineages of *E. faecalis*, including the vancomycin-resistant *E. faecalis* V583 strain, correlated with the lack of a functional CRISPR-Cas II system. CRISPR is an acronym for “clustered regularly interspaced short palindromic repeats,” and CRISPR consist of short repeat sequences interspersed with unique spacer sequences, with the spacer sequence often originating from known phages or plasmids (3, 4). A set of CRISPR-associated genes (*cas* genes) encoding nucleases are typically encoded near the CRISPR (5). The *E. faecalis* genome encodes the type II CRISPR-Cas locus, which consists of a CRISPR array, the type-specific *cas9* gene, and *cas1* and *cas2* genes (6, 7). Exposure to foreign DNA leads to the incorporation of a short segment (protospacer) of the invading MGE into the CRISPR which acts as a novel spacer in cells harboring the type II CRISPR-Cas system. By this mechanism, the CRISPR serves as a heritable genetic memory of encounters involving the foreign DNA. Subsequent encounters with MGEs harboring this conserved sequence motif result in a double-strand break in the DNA of an incoming MGE, thus serving as an effective adaptive immune mechanism that reduces the likelihood of a successful secondary encounter with foreign DNA (7).

Another mechanism by which *E. faecalis* strains, as well as many other bacteria, distinguish between “self” and “non-self” genetic material is the use of a restriction-modification (R-M) system. In this type of innate genetic defense, cells can recognize their “self” DNA based on specific methylation signatures and the “non-self” DNA (MGEs obtained via horizontal gene transfer) that lacks these methylation patterns is degraded by restriction endonucleases (8, 9). In a previous study, the Palmer laboratory identified an R-M system (EfaRF1) in *E. faecalis* strain OG1RF (10); importantly, only a subset of *E. faecalis* strains harbor an identifiable R-M system.

In their study, Price and colleagues experimentally confirmed the predicted role of the CRISPR3-Cas9 (type II CRISPR-Cas system) and the orphan CRISPR2 locus in the genome defense against pheromone-responsive plasmids (PRPs) (a type of MGE) (11). They have revealed the previously unreported synergism displayed between the CRISPR-Cas and the R-M system during genome defense against the acquisition of PRPs. The authors used the *E. faecalis* T11 strain to arrive at some of these important findings as T11 is closely related to the well-studied V583 strain but lacks many of the mobile genetic elements present in V583 and, importantly, possesses an intact R-M system and putative CRISPR3-Cas9 (12). In their initial experiment, the authors identified the *repB* sequence from the model pheromone-responsive pAD1 plasmid in spacer 6 of the T11 CRISPR3 locus. They deleted the *cas9* gene associated with the CRISPR3 locus in the T11 strain and showed a significant increase in the efficiency of pAD1 plasmid transfer via pheromone-mediated conjugation using a plate mating assay. The authors also showed that the CRISPR3 system provides genetic defense only against the acquisition of pAD1-like plasmids, since deletion of the CRISPR3-specific *cas9* gene did not affect the efficiency of mating with pCF10 (another well-studied PRP), presumably because T11 had not had a previous encounter with a pCF10-like plasmid. The individual deletion of the R-M system or the CRISPR3-specific *cas9* gene in the recipient T11 cells resulted in an ~150-fold increase in conjugation efficiency; however, when these two genome defense systems were deleted in tandem, the result was an ~4-log increase in conjugation efficiency for both a marked derivative of pAD1 (pAM714) and the pCF10 plasmids, suggestive of strong synergism between the two defense systems. The authors also shed light on the function of the orphan CRISPR2 system that is present in most MDR *E. faecalis* lineages but lacks dedicated Cas functions and demonstrated that this system requires CRISPR1-Cas-encoded factors to confer genome defense in the T11 strain (2, 10).

The acquisition of MGEs through horizontal gene transfer events has led to the emergence of pathogenic traits in many microbial pathogens, from diphtheria and cholera and their associated phages to the importance of plasmid biology in the pathogenesis of shigellosis, plague, and anthrax (13–15). Emergence of multidrug resistance in modern pathogens is no different and is driven by the acquisition of novel MGEs and has catalyzed the development of bacterial pathogens such as methicillin-resistant Staphylococcus aureus (MRSA), vancomycin-resistant *Staphylococcus aureus* (VRSA), and vancomycin-resistant *Enterococcus* (VRE) (16, 17). The consequence of functionally shutting down their CRISPR-Cas system or the R-M system could be a strategy to allow rapid acquisition of MGEs that in turn enables the newly emergent pathogen to efficiently survive environmental insults, including antibiotic treatment during infection. However, like a double-edged sword, the uncontrolled accretion of MGEs comes with a fitness cost, which is evident from the findings of a recent study by Gilmore et al. showing that the acquisition of MGEs by strain V583 confers a disadvantage to this strain in the absence of antibiotic selective pressure. The presence of two distinct MGEs in V583 renders it susceptible to an otherwise innocuous pheromone secreted by commensal enterococci (18). Further complicating genome defense strategies is a counteroffensive developed by some MGEs against the bacterial host. A study by Seed et al. showed that *Vibrio cholerae* is infected by a phage that possesses a phage-carried CRISPR/Cas system which is used by the phage to counteract a phage-inhibitory chromosomal island of the bacterial host (19). The absence of an R-M system in strain V583 is likely due to an allelic replacement by a conjugative transposon which carries vancomycin resistance, raising the issue of whether some MGEs direct movement into genome defense loci to ensure stable expression of MGE-carried genes. Understanding the factors that determine the outcome of this evolving molecular arms race between the bacterial host and the invading MGEs and whether the bacterial host chooses “to defend or not defend” its genome against invading MGEs could be critical for development of an effective strategy to curb infections caused by genetically promiscuous pathogens, such as MDR *E. faecalis*.
